# Laparoscopic Cholecystectomy for Acute Cholecystitis in a Patient with Acquired Hemophilia A: A Case Report of Perioperative Hemostatic Management with Plasma Exchange

**DOI:** 10.70352/scrj.cr.25-0533

**Published:** 2025-12-16

**Authors:** Koki Yasui, Hiroaki Mashima, Hisakazu Nishimori, Masashi Yoshimoto, Yuki Katsura, Fumitaka Taniguchi, Hiroyuki Sawada, Michihiro Ishida, Daisuke Sato, Ryuichi Yoshida, Yasuhiro Choda, Masanori Yoshimitsu, Kannyu Nakano, Yasuhiro Shirakawa, Hiroyoshi Matsukawa

**Affiliations:** 1 Department of Surgery, Hiroshima City Hiroshima Citizens Hospital, Hiroshima, Hiroshima, Japan; 2 Department of Hematology, Hiroshima City Hiroshima Citizens Hospital, Hiroshima, Hiroshima, Japan

**Keywords:** hemophilia, acquired hemophilia A, cholecystitis, acute cholecystitis, cholecystectomy, laparoscopic cholecystectomy, laparoscopic surgery, laparoscopy, plasma exchange

## Abstract

**INTRODUCTION:**

Acquired hemophilia A (AHA) is a rare but potentially life-threatening bleeding disorder caused by autoantibodies against coagulation factor VIII (FVIII). Surgery in patients with AHA is challenging because of the substantial risk of severe perioperative hemorrhage. However, no consensus has been reached on the optimal perioperative hemostatic strategies, especially for urgent surgical settings that require rapid correction of coagulation function, because of the rarity of this condition.

**CASE PRESENTATION:**

A 79-year-old woman was diagnosed with AHA and received immunosuppressive therapy (prednisolone and cyclophosphamide) and hemostatic treatment (activated prothrombin complex concentrate). She developed acute cholecystitis on day 23. However, surgery was initially contraindicated due to inadequate coagulation control, persistently prolonged activated partial thromboplastin time, low FVIII activity, and high FVIII inhibitor titer. Antibiotic therapy was initiated, and endoscopic nasobiliary gallbladder drainage was attempted on day 26. However, the procedure was unsuccessful. The worsening abdominal pain on day 27 prompted further interventions. Plasma exchange (PE) was performed to rapidly reduce the FVIII inhibitor titers and correct the coagulation status before laparoscopic cholecystectomy. The coagulation parameters markedly improved after 3 PE sessions, as evidenced by the shortened activated partial thromboplastin time, increased FVIII activity, and significant reduction in FVIII inhibitor titers. This enabled a safe laparoscopic cholecystectomy with minimal intraoperative blood loss on day 30. No additional PE was required, and the patient was discharged uneventfully on POD 16. The FVIII inhibitor was eliminated within 2 months, and immunosuppressive therapy was discontinued 4 months after surgery. The patient has been in remission to date.

**CONCLUSIONS:**

This case highlights the potential utility of PE as a bridging strategy to achieve rapid hemostatic correction in patients with AHA requiring urgent surgery, especially those with high-titer FVIII inhibitors. Immunosuppressive therapy and bypassing agents remain the cornerstones of AHA management. However, PE may be an effective adjunct treatment option for refractory cases or in time-constrained settings. This report provides valuable insights into individualized multidisciplinary approaches that may facilitate safe surgical intervention in patients with AHA.

## Abbreviations

ACT
: activated clotting time
AHA
: acquired hemophilia A
aPCC
: activated prothrombin complex concentrate
aPTT
: activated partial thromboplastin time
BU
: Bethesda units
CPA
: cyclophosphamide
FVIII
: coagulation factor VIII
PSL
: prednisolone
PE
: plasma exchange
rFVIIa
: recombinant activated factor VII
vWF
: von Willebrand factor

## INTRODUCTION

AHA is a rare but potentially life-threatening bleeding disorder caused by autoantibodies that neutralize FVIII.^1)^ The management of AHA, which involves immunosuppressive therapy to eradicate the FVIII inhibitor and hemostatic treatment using bypassing agents to control active bleeding, is well established.^2–5)^ However, surgical intervention for patients with AHA remains challenging because of the high risk of perioperative hemorrhagic complications. Moreover, the rarity of this condition has precluded the establishment of evidence or consensus on optimal perioperative management of bleeding in patients with AHA, especially in the context of emergent or urgent surgery.^3)^ We report the case of a patient with AHA who developed acute cholecystitis despite disease control with immunosuppressive therapy and bypassing agents. PE promptly eliminated the circulating FVIII inhibitors and improved the coagulation status of the patient. This allowed an urgent laparoscopic cholecystectomy without hemorrhagic complications.

## CASE PRESENTATION

**Fig. 1:**
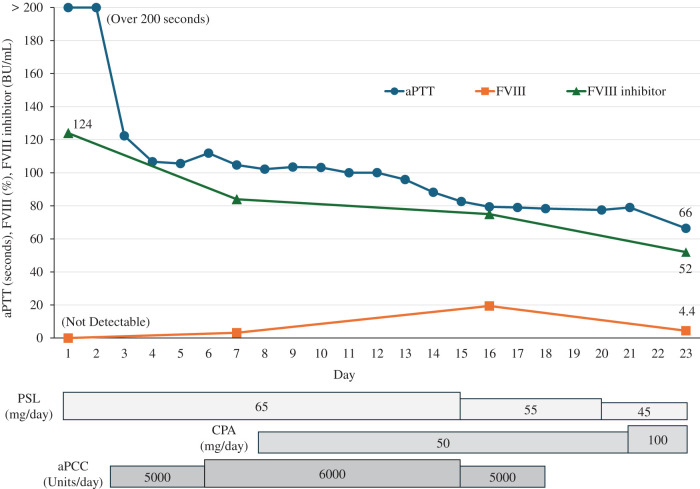
Clinical course from the diagnosis of acquired hemophilia A to the onset of acute cholecystitis. The upper panel shows the clinical course of the aPTT, FVIII activity, and FVIII inhibitor titers. The lower panel shows the treatments for acquired hemophilia A. aPCC, activated prothrombin complex concentrate; aPTT, activated partial thromboplastin time; CPA, cyclophosphamide; FVIII, coagulation factor VIII; PSL, prednisolone

**Fig. 2:**
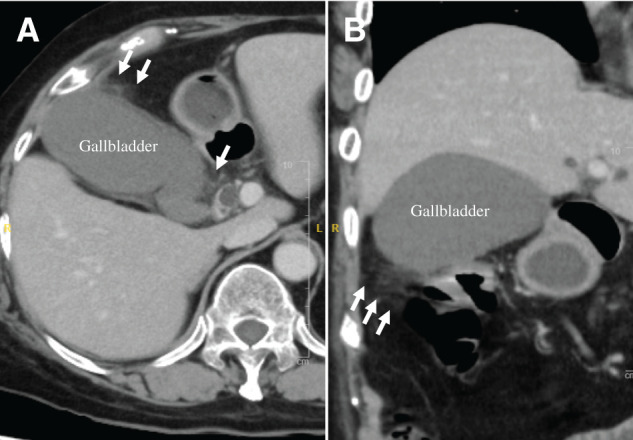
Abdominal contrast-enhanced CT findings. CECT shows gallbladder distension and pericholecystic inflammatory changes (arrows). The gallbladder wall shows no contrast enhancement, indicating gangrenous cholecystitis. (**A**) Axial image. (**B**) Coronal image. CECT, contrast-enhanced CT

**Fig. 3:**
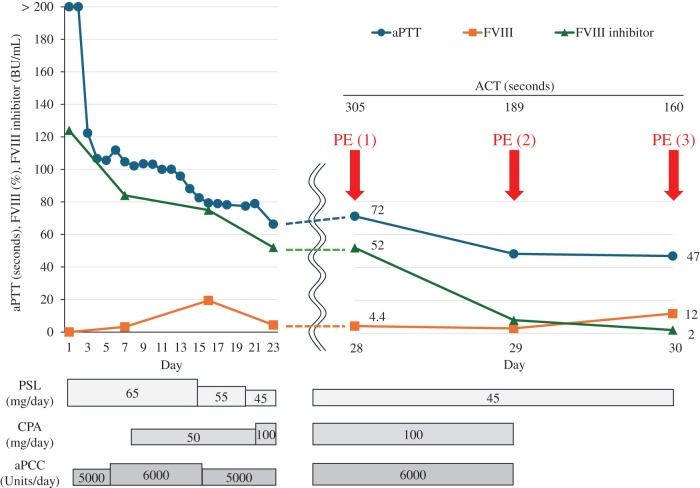
Changes in coagulation function during preoperative PE sessions. The coagulation parameters improved rapidly after 3 PE sessions, and the patient underwent laparoscopic cholecystectomy on day 30. ACT, activated clotting time; aPCC, activated prothrombin complex concentrate; aPTT, activated partial thromboplastin time; CPA, cyclophosphamide; FVIII, coagulation factor VIII; PE, plasma exchange; PSL, prednisolone

**Fig. 4:**
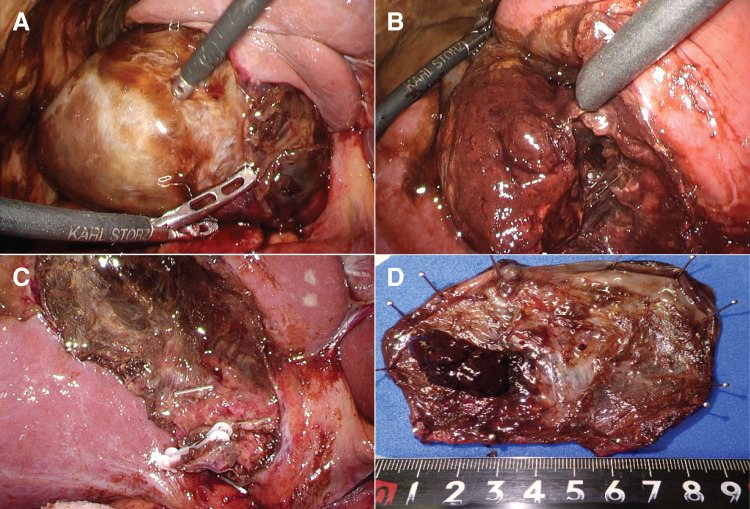
Intraoperative findings. Gangrenous cholecystitis with bile leakage and localized peritonitis was also identified. Standard laparoscopic cholecystectomy was performed with minimal blood loss. The duration of surgery was 139 min. (**A**, **B**) Gallbladder appearance. (**C**) Gallbladder bed after completion of resection. (**D**) Resected specimen.

**Fig. 5:**
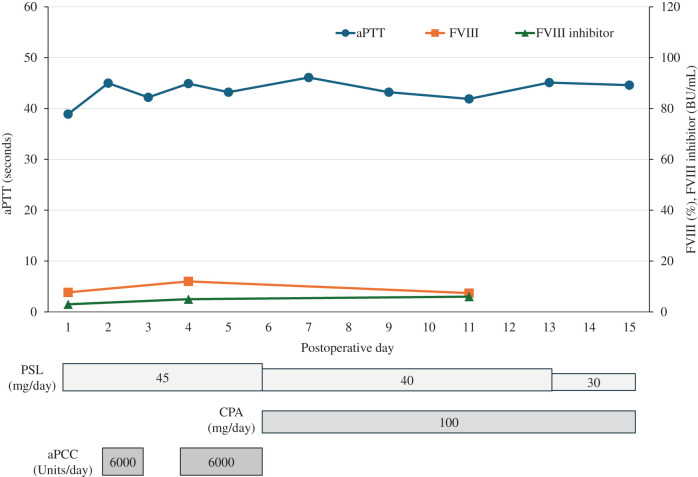
Postoperative course (from surgery to hospital discharge). Coagulation function was maintained without additional plasma exchange after surgery. The patient was discharged uneventfully on POD 16. aPCC, activated prothrombin complex concentrate; aPTT, activated partial thromboplastin time; CPA, cyclophosphamide; FVIII, coagulation factor VIII; PSL, prednisolone

**Fig. 6:**
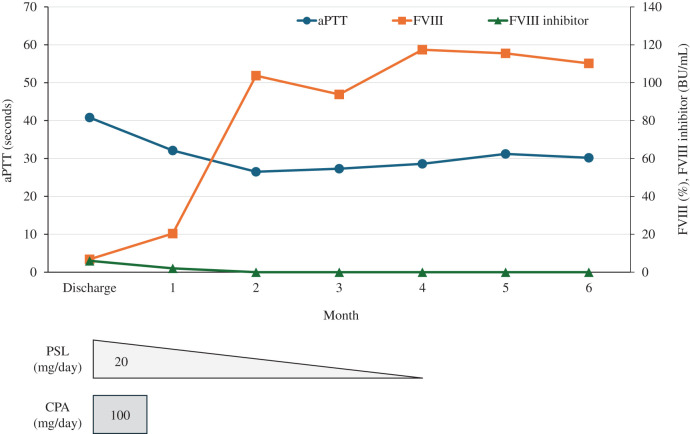
Clinical course of acquired hemophilia A after hospital discharge. The FVIII inhibitor was undetectable, and the aPTT and FVIII activity had normalized at 2 months after surgery. Immunosuppressive therapy was discontinued 4 months postoperatively. aPTT, activated partial thromboplastin time; CPA, cyclophosphamide; FVIII, coagulation factor VIII; PSL, prednisolone

A 79-year-old female presented with a thigh hematoma, subcutaneous bleeding in both hands, and hematuria. She had no history of anticoagulant or antiplatelet use or personal or family history of bleeding disorders. The aPTT was markedly prolonged (>200 s). The prothrombin time and platelet count were normal, and the test for lupus anticoagulants was negative (**Table 1**). The cross-mixing test failed to correct the aPTT, suggesting the presence of an inhibitor. Subsequent coagulation factor assays revealed undetectable FVIII activity (<0.9%) and a high-titer FVIII inhibitor (124 BU/mL). The vWF antigen and activity were normal (**Table 1**). Immunosuppressive therapy with PSL (65 mg/day [1 mg/kg]) and CPA (50 mg/day) was initiated based on the diagnosis of AHA. aPCC was administered concomitantly for hemostatic control. The bleeding symptoms and laboratory findings gradually improved with these treatments (**Fig. 1**). However, the patient developed upper abdominal pain and was diagnosed with acute cholecystitis on hospital day 23 (**Fig. 2**). The aPTT remained prolonged (66 s), and the FVIII activity was persistently low (4.4%) with a high titer of FVIII inhibitor (52 BU/mL) (**Fig. 1**). Therefore, invasive interventions, including cholecystectomy and percutaneous transhepatic gallbladder drainage, were deemed inappropriate at that time, and antibiotic therapy was initiated for the management of acute cholecystitis. Endoscopic nasobiliary gallbladder drainage was attempted on hospital day 26. However, endoscopic nasobiliary drainage was performed instead because cannulation of the gallbladder was unsuccessful. Nevertheless, the patient developed worsening abdominal pain on day 27, which indicated the need for further intervention, including cholecystectomy. Although delaying surgery entailed a risk of infection progression, the potential danger of catastrophic bleeding was judged to outweigh that risk. Therefore, PE was initiated to rapidly eliminate the FVIII inhibitors and correct coagulation function before laparoscopic cholecystectomy. After 3 PE sessions, the aPTT improved to 47 s, FVIII activity increased to 12%, the FVIII inhibitor titer decreased to 2 BU/mL, and ACT improved from 305 to 160 s (**Fig. 3**). As these results indicated that coagulation function had recovered to a minimally acceptable level for surgery, the patient underwent laparoscopic cholecystectomy on hospital day 30. The intraoperative findings revealed a completely gangrenous gallbladder accompanied by bile leakage and localized peritonitis (**Fig. 4**). However, no uncontrollable intraoperative bleeding was observed, and standard cholecystectomy was successfully performed with minimal blood loss. aPCC was administered on PODs 2, 4, and 5, but no additional PE was required (**Fig. 5**). The postoperative course was uneventful, and the patient was discharged on POD 16. The FVIII inhibitor had been eliminated, and both the aPTT and FVIII activity had normalized by the 2-month follow-up. Immunosuppressive therapy was discontinued at 4 months after surgery, and remission has been maintained (**Fig. 6**).

**Table 1:** Laboratory findings at diagnosis of acquired hemophilia A

Parameter	Result	Parameter	Result
WBC	9.8 × 10^4^/μL	PT	98.6%
RBC	380 × 10^4^/μL	PT-INR	1.01
Hb	12.1 g/dL	aPTT	68.4 s
Hct	36.9%	Fibrinogen	671 mg/dL
Plt	23.3 × 10^4^/μL	FDP	18.6 μg/mL
T-Bil	0.4 mg/dL	D-dimer	12.6 μg/mL
AST	18 U/L	FVIII activity	<0.9%
ALT	13 U/L	FVIII inhibitor	124 BU/mL
LDH	236 U/L	FIX activity	109.6%
γ-GTP	34 U/L	FIX inhibitor	ND BU/mL
Alb	3.1 g/dL	vWF activity	317%
BUN	18 mg/dL	Lupus anticoagulant	1.1
Cre	0.76 mg/dL		
CRP	7.19 mg/dL		

Alb, albumin; ALT, alanine aminotransferase; aPTT, activated partial thromboplastin time; AST, aspartate aminotransferase; BU, Bethesda units; BUN, blood urea nitrogen; Cre, creatinine; CRP, C-reactive protein; FDP, fibrin/fibrinogen degradation products; FIX, coagulation factor IX; FVIII, coagulation factor VIII; Hb, hemoglobin; Hct, hematocrit; INR, international normalized ratio; LDH, lactate dehydrogenase; ND, not detected; Plt, platelet count; PT, prothrombin time; RBC, red blood cell; T-Bil, total bilirubin; vWF, von Willebrand factor; WBC, white blood cell; γ-GTP, gamma-glutamyl transpeptidase

## DISCUSSION

AHA is a rare condition with an estimated incidence of 1–1.5 cases per million population per year. It predominantly affects older adults with malignancies or autoimmune diseases and in postpartum women.^2,3,5)^ AHA should be suspected in patients with spontaneous bleeding who have no history of trauma or previous bleeding disorders, especially if they have isolated prolongation of aPTT.^4)^ AHA was 1st identified in some cases after unexpected perioperative hemorrhagic complications.^2,4)^ Therefore, surgeons should be aware of this condition. Although AHA is primarily an autoimmune disorder, infections or inflammatory conditions such as cholecystitis have been reported as potential triggers or exacerbating factors for autoantibody production. However, in the present case, AHA was diagnosed before the onset of acute cholecystitis; thus, the 2 conditions were most likely coincidental.

The standard treatment involves immunosuppressive agents such as PSL, CPA, and rituximab to eradicate FVIII inhibitors and hemostatic therapy using bypassing agents such as rFVIIa or aPCC, which activate the coagulation cascade independently of FVIII.^1–6)^ Recombinant porcine factor VIIIa has recently emerged as another effective treatment option for patients with severe bleeding.^1,3)^ However, there is currently no standardized strategy for safe perioperative hemostasis in patients with AHA undergoing surgical procedures. The American Society of Hematology recommends the use of aPCC or rFVIIa for perioperative hemostasis,^7)^ and previous reports have demonstrated the successful management of patients after elective pulmonary, cardiovascular, and gastrointestinal surgeries for AHA using intensive perioperative administration of bypassing agents in conjunction with immunosuppressive therapies.^8–11)^ Surgery was required urgently in our case, despite the high FVIII inhibitor titer and impaired coagulation. To the best of our knowledge, only a few reports have described this situation.^10)^

PE is not part of the standard treatment strategy. However, it may be an effective adjunctive therapy for patients with AHA, especially those with high-titer FVIII inhibitors who are refractory to immunosuppressive therapy or for whom such therapy is contraindicated due to active infectious complications.^12,13)^ PE can rapidly eliminate circulating FVIII inhibitors and facilitate hemostasis. PE can also supplement depleted coagulation factors through replacement fluids such as fresh frozen plasma. It may also be considered a cost-effective alternative treatment given the limited availability and high cost of bypassing agents.^14)^ Several case reports have documented clinical improvements following 2 to 8 sessions of PE in combination with immunosuppressive therapies. These improvements include normalization of aPTT and FVIII activity, reduction in inhibitor titers, and resolution of bleeding symptoms.^12–14)^ However, PE has not been reported as a preparatory measure for emergency or urgent surgery in patients with AHA.

The hemostatic condition of the patient was inadequate for safe laparoscopic cholecystectomy at the time of cholecystitis onset despite intensive immunosuppressive therapy with PSL and CPA and administration of aPCC as hemostatic treatment (**Fig. 1**). Rituximab was considered but deemed unsuitable as a perioperative treatment for urgent surgery because its therapeutic effect requires several weeks to achieve adequate control of AHA.^4,6)^ Additional use of rFVIIa was also considered; however, its use was restricted at our institution and therefore could not be implemented. Given the persistently high FVIII inhibitor titer and these limitations, PE was selected as a bridging and adjunctive strategy to achieve rapid preoperative disease control. The aPTT, FVIII activity, FVIII inhibitor titer, and ACT decreased from 72 to 47 s, 4.4 to 12%, 52 to 2 BU/mL, and 305 to 160 s, respectively, after 3 PE sessions (**Fig. 3**). There are no validated laboratory thresholds to determine surgical readiness in AHA. However, according to the World Federation of Hemophilia guidelines for congenital hemophilia, preoperative FVIII levels of 50%–80% are recommended for minor procedures and 80%–100% for major surgeries.^15)^ In the present case, although the FVIII activity remained suboptimal after PE, both the aPTT and ACT improved to levels considered acceptable for proceeding with surgery under careful perioperative hemostatic control. Consequently, laparoscopic cholecystectomy was safely performed without perioperative hemorrhagic or infectious complications. The FVIII inhibitor concentration did not increase after surgery, and the AHA remained well controlled without additional PE (**Fig. 5**). A previous report of 6 cases with high-titer FVIII inhibitors suggested that PE may not only be effective for temporarily removing FVIII inhibitors but also for facilitating the remission of AHA under adequate immunosuppressive therapy.^14)^ These findings indicate that PE may play a supportive role in the management of AHA, particularly in patients with high-titer FVIII inhibitors who are unresponsive to standard treatments. Therefore, PE should be regarded as an adjunctive measure that can enhance the efficacy of conventional immunosuppressive and hemostatic therapies.

In addition to the aforementioned benefits, it should be noted that the PE procedure itself requires invasive vascular access and anticoagulation, both of which may increase the risk of bleeding, particularly in patients with marked coagulation dysfunction. Several reports have documented procedural or periprocedural bleeding complications associated with PE in patients with hemophilia, underscoring the importance of continuous monitoring and individualized risk assessment. Therefore, although PE may facilitate urgent surgical intervention, its inherent bleeding risks must be carefully weighed against its therapeutic advantages and managed through meticulous perioperative planning and close multidisciplinary collaboration.^13,16)^

## CONCLUSIONS

The perioperative management of AHA, especially for cases requiring urgent surgical intervention, is not standardized. This case highlights the potential role of PE as a bridging and supportive strategy in combination with immunosuppressive therapy and bypassing agents. PE may be a useful adjunct for rapidly reducing FVIII inhibitor titers and stabilizing coagulation to enable safe surgical procedures. This is a single case report, and the effectiveness of PE in similar situations remains unclear. Further studies are needed to determine the optimal timing, indications, and number of PE sessions for the perioperative management of AHA.

## DECLARATIONS

### Availability of data and materials

All data generated or analyzed during this study are included in this article.
